# Phylogenetic Analysis of ORF Viruses From Five Contagious Ecthyma Outbreaks in Argentinian Goats

**DOI:** 10.3389/fvets.2018.00134

**Published:** 2018-06-19

**Authors:** Andrea Peralta, Carlos A. Robles, Juan F. Micheluod, Carlos E. Rossanigo, Agustín Martinez, Agustín Carosio, Guido A. König

**Affiliations:** ^1^Instituto de Biotecnología, Centro Nacional de Investigaciones Agropecuarias, Instituto Nacional de Tecnología Agropecuaria, Consejo Nacional de Investigaciones Científicas y Técnicas, Buenos Aires, Argentina; ^2^Grupo de Sanidad Animal, Instituto Nacional de Tecnología Agropecuaria, Bariloche, Argentina; ^3^Grupo de Sanidad Animal, Instituto de Investigación Animal del Chaco Semiárido, Centro de Investigaciones Agropecuarias, Instituto Nacional de Tecnología Agropecuaria, Salta, Argentina; ^4^Grupo de Sanidad Animal, Instituto Nacional de Tecnología Agropecuaria, San Luis, Argentina

**Keywords:** Contagious Ecthyma, Orf virus, goats, molecular epidemiology, Parapoxvirus

## Abstract

Orf virus (ORFV) is the etiological agent of Contagious Ecthyma (CE) disease that mainly affects sheep, goats, wild ruminants, and humans with a worldwide distribution. To date, only two strains from Argentinian sheep have been characterized at the molecular level and there is little information on ORFV strains circulating in Argentina. Here we describe and analyze five outbreaks of CE in goats in three geographic regions of the country: Northwest, Center, and Southwest. The phylogenetic analysis based on four molecular markers of ORFV (*orf011* partial sequence and *orf020, orf109*, and *orf127* complete sequence genes) revealed that there are different strains circulating in Argentina and pointed out the importance of knowing the health status of animals traded between farms.

## Introduction

Contagious Ecthyma (CE) is a viral skin disease (contagious pustular dermatitis) that mainly affects sheep and goats, but can also affect other ruminants and wild animals (1–4). CE is also a zoonosis of global distribution that affects humans, particularly animal workers, like slaughtermen, veterinarians, farmers, and animal caretakers, after direct or indirect contact with infected animals (5, 6).

CE is caused by the Orf virus (ORFV), an epitheliotropic virus that commonly produces lesions on the lips, oral and nasal mucosa, skin, and udders (1). These lesions evolve through different stages such as erythema, macules, vesicles, pustules, and proliferative scabs (7). The lesions are painful and can lead to anorexia and starvation (8). Although the disease is relatively benign, the outbreaks generate economic losses associated with a growth delay and deterioration of the animal's body condition. The virus is extremely resistant to desiccation and can remain for many years in dry crusts of animals that suffered the disease.

ORFV is a prototype member of Parapoxvirus (PPV) genus which belongs to the *Poxviridae* family. It has a linear double-stranded DNA genome, of ~135 kbp in size and exhibits an unusually high GC content (~64%). The central region of the genome contains 88 genes which are present in the subfamily *Chordopoxvirinae* and mostly occur in a common order and orientation. The terminal regions of the genome are variable and there are genes associated with virulence, pathogenesis, tropism, and/or immune response modulators, such as *orf020, orf112, orf117, orf127*, and *orf132* (7, 9–11).

In Argentina, veterinarians have detected outbreaks of CE in all geographical regions by recognizing clinical manifestation of disease (12, 13). Although a live Ecthyma vaccine is used in some Argentinian regions, genetic data regarding this vaccine is scarce (14).

In South America, there were recently described CE outbreaks in Argentina, Brazil, and Uruguay. A molecular characterization of *orf011* gene (partial sequence) is available in ORFV strains from Brazil (14, 15) and Uruguay (16), whereas in Argentina, only two ORFV isolates from sheep were characterized at the molecular level (17).

The objective of this work was to identify and molecularly characterize the Orf virus that caused five CE outbreaks in goats in three geographic regions of Argentina during the period 2014–2015.

## Materials and methods

### Origin of samples

**Figure 1:**
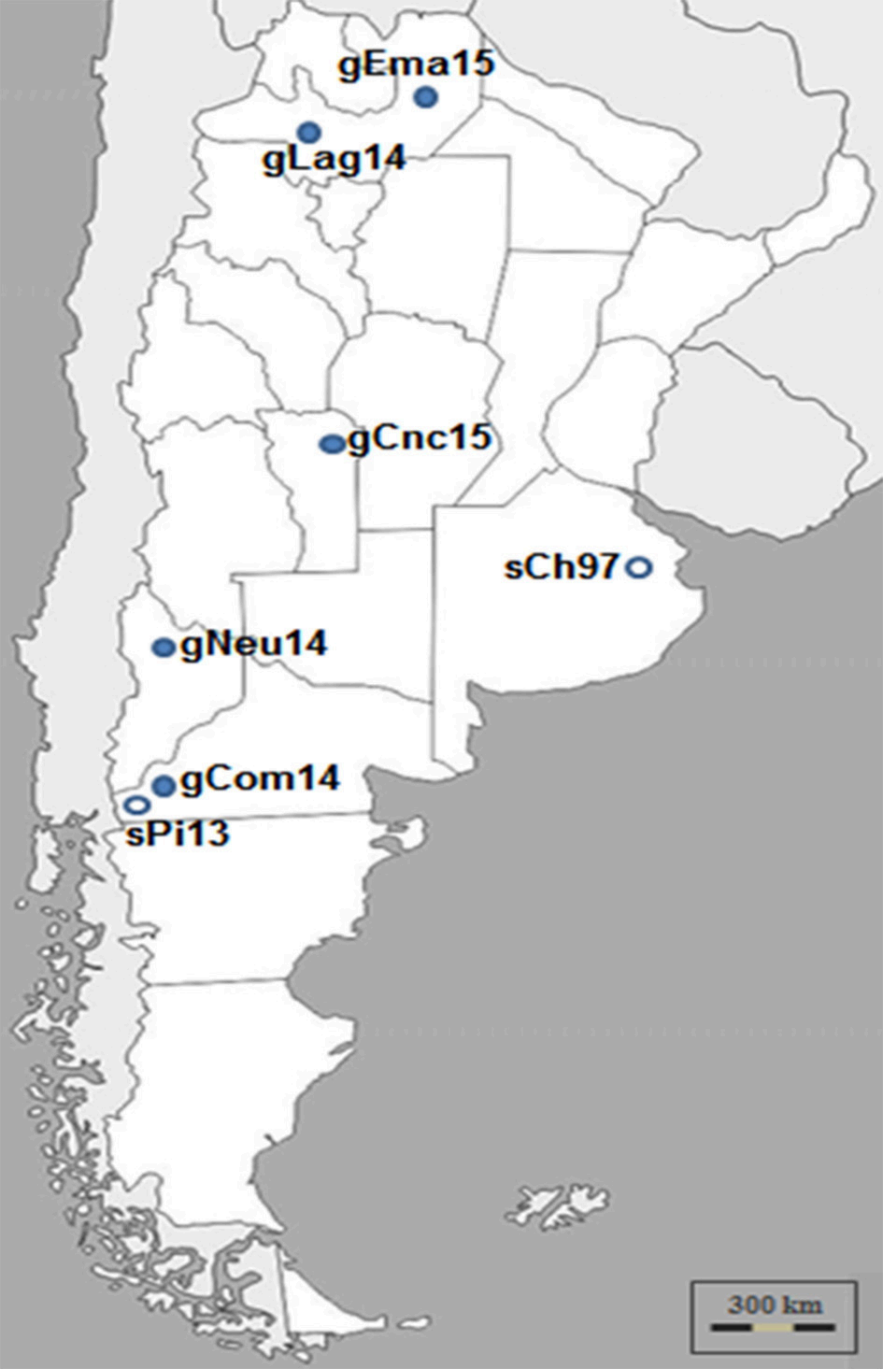
Argentinian ORFV analyzed in this study. ORFV samples were obtained during Contagious Ecthyma outbreaks from different regions of Argentina and time points. Dark circles indicate Ecthyma outbreaks occurred in goats while white circles outbreaks in sheep (17).

Samples from five outbreaks of clinical CE that occurred in three different geographic regions of Argentina: Northwest, Center, and Southwest, during 2014 and 2015 were analyzed (Figure 1).

The samples from Patagonia, the southern region of the country, belonged to Pichi-Neuquén (36°62′S 70°81′W, Neuquén province) and Comallo (41°03′S 70°27′W, Río Negro province). The samples from the northern region of the country were taken from La Aguadita (25°21′S 66°17′W) and El Manantial (24°24′S 63°41′W), both locations situated in Salta province. Finally, samples belonging to a goat flock from Concarán (32°55′S 65°25′W, San Luis province) were taken in the central region of the country. The samples were called gNeu14, gCom14, gLag14, gEma15, and gCnc15, respectively.

Veterinarians, who attended the outbreaks, recorded the number and age of affected animals, the severity of injuries, movement of animals, etc. Scabs were collected using scalped blades and tweezers, and the samples were stored at refrigerated temperatures until arrival to the laboratory, and subsequently at −80°C until processing.

### Electron microscopy

Five hundred milligrams (500 mg) of scab material were macerated in mortar under liquid nitrogen until a homogeneous powder was obtained. A 30% suspension (weight/volume) was made in TMN buffer (10 mM Tris-HCl pH 7.5; 1.5 mM MgCl_2_; 10 mM NaCl), freeze-thawed at −70°C once and sonicated three cycles of 2 min each, in a bath (Elmasonic, sweep mode). Then, the suspension was centrifuged at 2,000 × g for 10 min at 4°C. The clarified supernatant was loaded onto a 30% sucrose cushion (30% w/w sucrose in TMN buffer) and pelleted for 70 min at 42,000 × g. Pellets were resuspended in TMN buffer, negatively stained with 2% phosphotungstic acid and then examined on a 200 mesh grid, under the transmission electron microscope (Zeiss EM109T at 85 kV). Transmission electron microscopy observation was carried out at the National Microscopy Service, School of Medicine, Buenos Aires National University.

### Extraction of genomic DNA

Total DNA was extracted from the collected scabs by using QIAamp DNA Mini kit (QIAGEN) according to the manufacturer's instructions. Briefly, the sample scabs were thoroughly homogenized in liquid nitrogen and 20 mg of the resulting tissue powder was placed in a microcentrifuge tube and incubated with lysis buffer and proteinase K at 56°C in a water bath until complete tissue lysis. DNA was then extracted, according to the manufacturer's instructions, eluted with 0.2 mL of elution buffer and finally stored at −20°C.

### PCR reactions and sequencing of ORFV genome regions

For ORFV, detection primers 045F and 045R were used following the protocol established by Kottaridi et al. (18). These oligonucleotides amplify an internal region from the *orf045* gene [nucleotide (nt) position 44–435].

After confirming the presence of the viral genome, we used the DNA samples as a template for PCR reactions to amplify an *orf011* gene fragment and the complete open reading frame for *orf020, orf109*, and *orf127* genes, as described in Peralta et al. (17). Briefly, each reaction mixture (total volume: 50 μL) contained 10 μL 5 × GoTaq green buffer, 1 μL 10 mM dNTPs mix, 0,2 μL GoTaq DNA polymerase (Promega), 100 ng of extracted DNA, and 25 pmoles of specifics primers.

PCR products were resolved by electrophoresis in 1.2% agarose gel, stained with Ethidium Bromide (10 mg/mL), and then visualized under UV light.

The PCR fragments were directly sequenced in both orientations, by using the amplification primers. The four amplicon sequences were directly obtained with the Big Dye terminator kit TM (Applied Biosystems, Foster City, CA, USA) in an ABI 3500 XL TM (Applied Biosystems, Foster City, CA, USA). Genomic information was derived from overlapping sequences covered by the forward and reverse primers. The sequences were annotated in the GenBank (accession numbers in Table 1).

**Table 1:** Gen Bank accession number of Argentinian ORFV strains.

	***orf011* (Partial sequence)**	***orf020***	***orf109***	***orf127***
gNeu14	KY863423	KY863428	KY863433	KY863438
gCom14	KY863424	KY863429	KY863434	KY863439
gSal14	KY863425	KY863430	KY863435	KY863440
gEma15	KY863426	KY863431	KY863436	KY863441
gCnc15	KY863427	KY863432	KY863437	KY863442

### Recombination and phylogenetic analyses

The sequences were aligned with previously published PPV sequences from GenBank by using the Clustal W method (19), and then manually refined alignments were performed using BioEdit software (20).

For recombination analysis, the four genome regions of all five Argentinean goat isolates, the two Argentinean sheep isolates and the three reference strains, were concatenated in a single 2,059 nucleotide alignment and the analysis was performed with RDP4 software, v4.95 (21). Phylogenetic trees were constructed using the Neighbor-joining Method with 1,000 bootstrap replicates implemented by MEGA software version 6 (22). The analysis of the evolution model was also evaluated with this program and Kimura-2 parameters (23) were selected for *orf127* gene while for the others analyzed sequences the Tamura-3 parameters evolution model (24) was used.

The Parsimony method, implemented by Mega6 software, was also used in the analysis of the *orf011* internal region.

## Results

### Cases presentation

**Figure 2:**
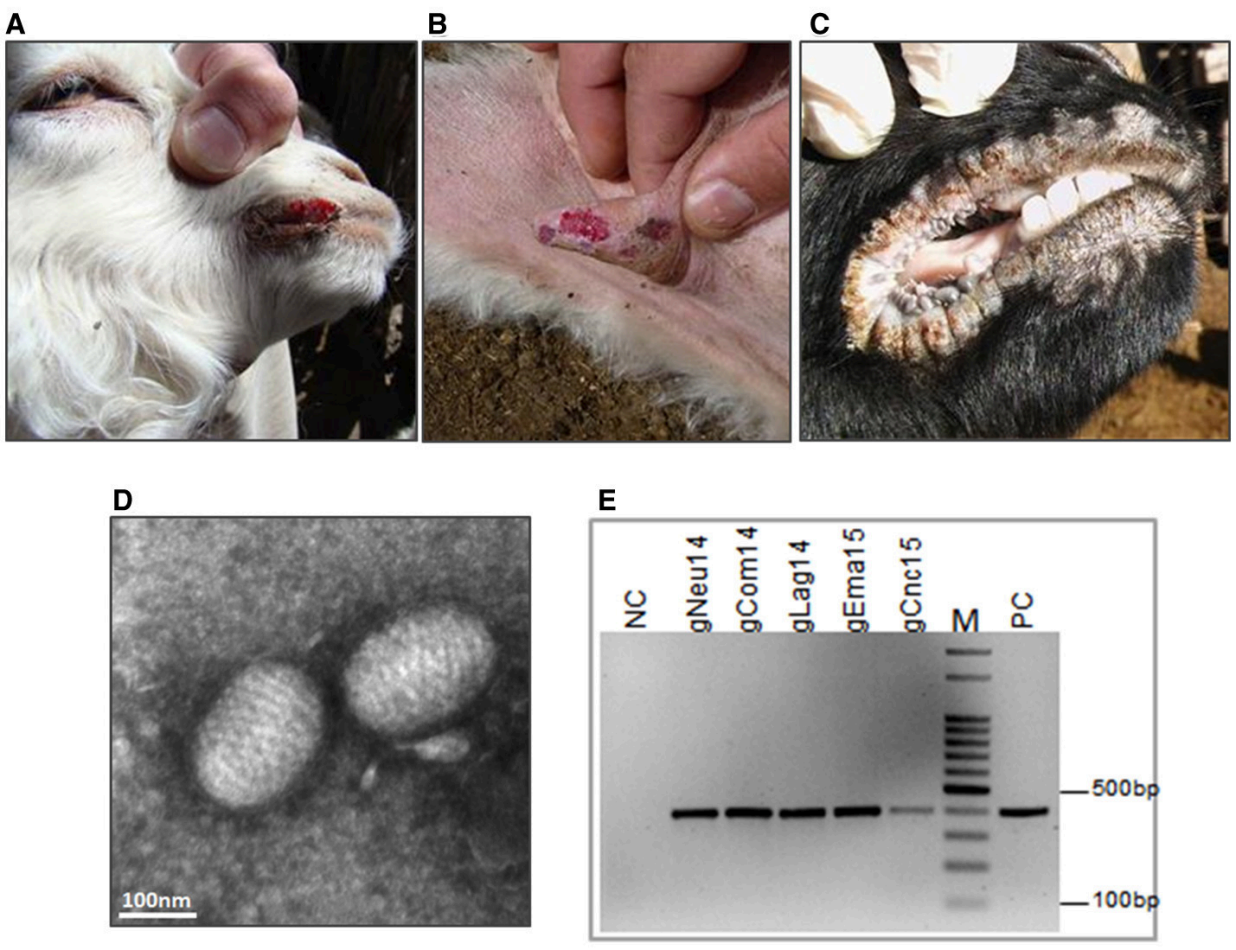
Ecthyma outbreaks: clinical diagnosis and molecular confirmation. Contagious Ecthyma in Angora goats from Comallo outbreak: lesions in lips **(A)** and udder **(B)**. **(C)** Lesions in lips from Creole goats from the Concaran outbreak. **(D)** Electron microphotograph showing the characteristic morphology of a parapoxvirus virion from El Manantial scabs (bar = 100 nm). **(E)** Confirmatory PCR, detection of ORFV genome in all analyzed samples. NC, negative control; PC, positive control; M, DNA ladder 100 bp.

In 2014, Ecthyma outbreaks were detected in two goat flocks from the Southwestern region of Argentina. The first outbreak was in a farm near Pichi-Neuquén place. The sample corresponds to an 8-month-old Creole kid showing typical lesions of CE with proliferative crust lesions on the lips and nostrils. At the time of the sampling (May 2014), this was the only animal affected out of 350 mothers and 150 kids. The second case was caused by a group of asymptomatic Creole goats from Neuquén province that entered in contact with an Angora goat flock in Comallo Town (Río Negro province) in September 2014. Only a few days after, the last ones started to present EC symptoms. The incidence was high (34%) and the affected animals, mainly pregnant or lactating goats, presented crusty lesions in the udder, mouth and foot area of anterior and posterior limbs (Figures 2 A,B).

Additionally, we analyzed two CE outbreaks from the northwestern region of the country. The first was in La Aguadita place in November 2014. This outbreak affected a dairy flock of Saanen goats. Young animals were most affected presenting lesions concentrated around the mouth. The incidence was ~20%, and it is suspected that the disease entered the flock after incorporating adult goats 30 days before symptoms detection.

The second outbreak occurred in a small flock in El Manantial place, in May 2015. The lesions, although severe, were restricted to the mouth. The incidence was low (10%) and only six adults were affected. These animals had entered the herd about 20 days before the outbreak started.

Finally, in August 2015, a CE outbreak was detected in a flock in the town of Concarán, at the central region of the country, at the mating season. The disease affected two adult animals (one male and one female goat) out of 50 and two kids out of 20. In all cases, the lesions were limited to mouth and lips (Figure 2C).

### Electron microscopy and confirmatory PCR

Transmission electron microscopic examination of the processed scabs revealed the presence of negatively stained oval-shape virus particles with the arrangement of the protein filaments (Figure 2D and Figure S3). The size of the virion was approximately 140 × 250 nm. To confirm the ORFV identity, total DNA was extracted from the scabs samples and screened by PCR amplification. A fragment of 392 bp was amplified from all samples (Figure 2E and Figure S4).

### Phylogenetic analysis

We selected four different genome regions of interest to perform a phylogenetic analysis. For this purpose, we amplified these sequences by PCR and subsequently sequenced them.

First, we obtained a specific PCR product of 594 bp from the *orf011*gene (25) from all samples. This gene is commonly used for OFRV phylogenetic analysis (14, 26, 27). The nucleotide (nt) identity among Argentinian goats samples was 98.3–99.8%. Particularly, the gNeu14 strain was the most similar to the Argentinian sheep samples previously described sPi13 and sCh97 (17) with an identity value of 99.6 and 99.4%, respectively.

**Figure 3:**
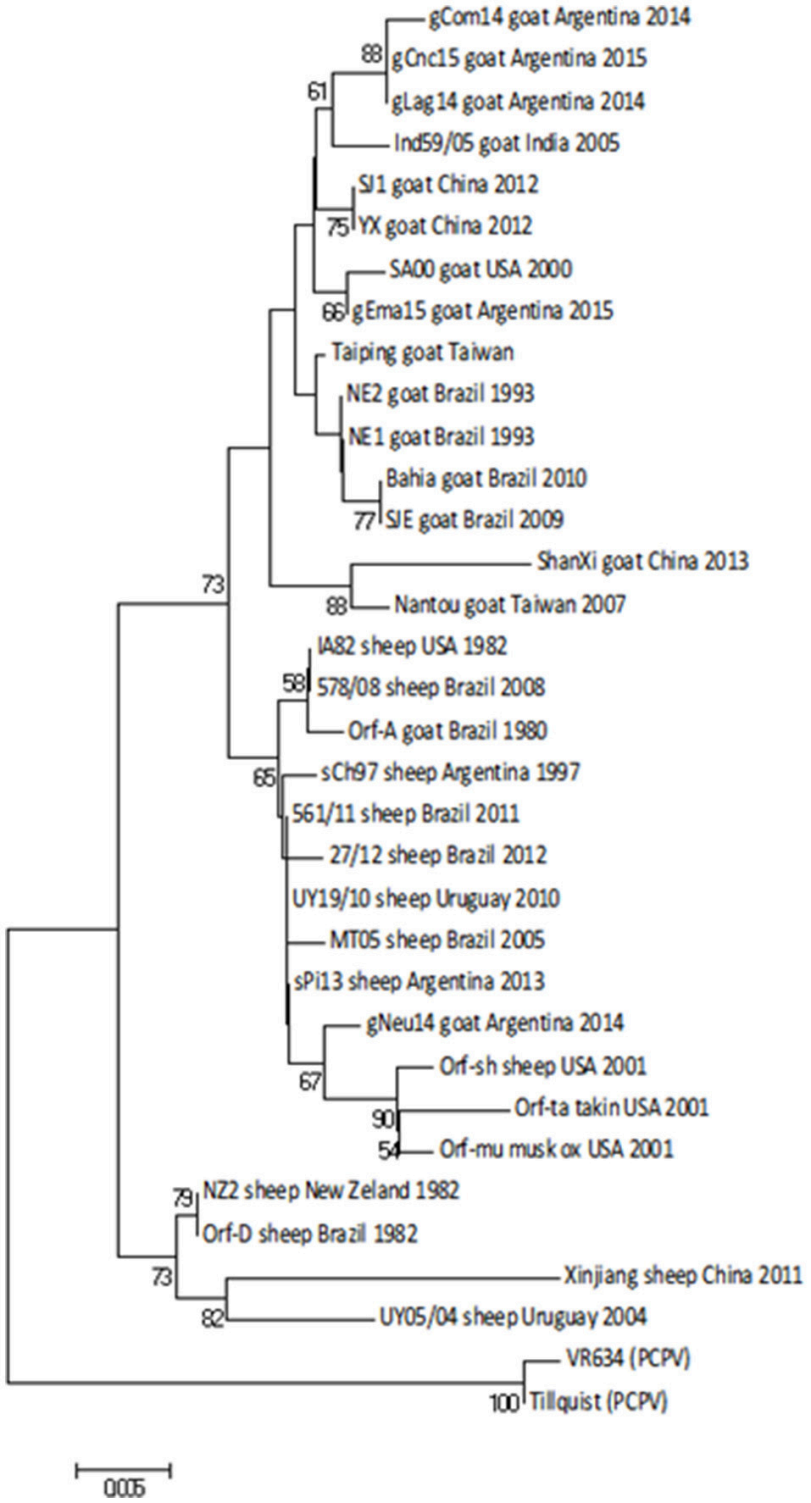
Phylogenetic analysis based on nucleotide sequence of *orf011*. The phylogenetic relationship was constructed by the Neighbor-Joining algorithm using MEGA 6.0 software. Numbers at nodes represent % of one thousand bootstrap replicates. The scale bars are expressed in relative nucleotide sequence difference.

Then we constructed a Neighbor-Joining (NJ) phylogenetic tree using the Tamura-3 parameters as evolution model. In this tree, gCom14, gCnc15, and gLag14 formed a group with a high bootstrap confidence value of 88% (Figure 3) and they are part of a larger group formed exclusively by strains from goats. As we expected, the gNeu14 strain is in the same group than the Argentinian sheep strain sPi13 and sCh97 and others South or North American strains (Orf-A, MT05, 578/08, 561/11, 27/12, UY19/10, IA82, Orf-mu, Orf-ta, Orf-sh). Most of them correspond to strains coming from sheep.

**Figure 4:**
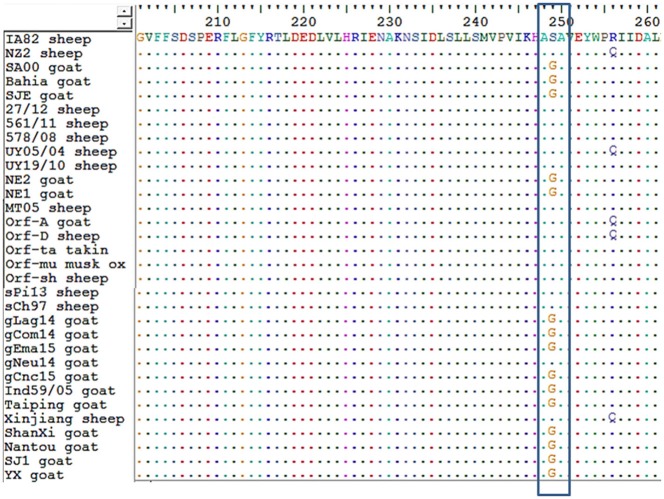
Alignment of amino acid sequences from *orf011*. An alignment of amino acid sequences was performed for all strains of ORFV analyzed in the *orf011* phylogenetic tree. In the box, position 249 is shown.

Regarding amino acid sequences, Kumar et al. (28), described a serine at position 249 as a species marker. Our alignment also showed this *orf011* S249 in ORFV strains from sheep. Besides, we observed that ORFV strains from goats grouping together with ovine strains also have a serine in position 249 (Figure 4).

In order to improve the analysis, we extended the study to other ORFV genes.

The identity matrix of the nt sequences from *orf020* (*VIR gene*) revealed a nucleotide identity of 95.3–99.8% among Argentinian goat strains. Similar to the *orf011*gene, the gNeu14 strain was more related to Argentinian sheep strains sPi13 and sCh97 with an identity value of 98.2 and 97.7%, respectively.

The range of nt identity among all the reported strains from Argentina is 94.6–99.5% and it is between the value obtained for all *orf020* gene sequences deposited in GenBank.

**Figure 5:**
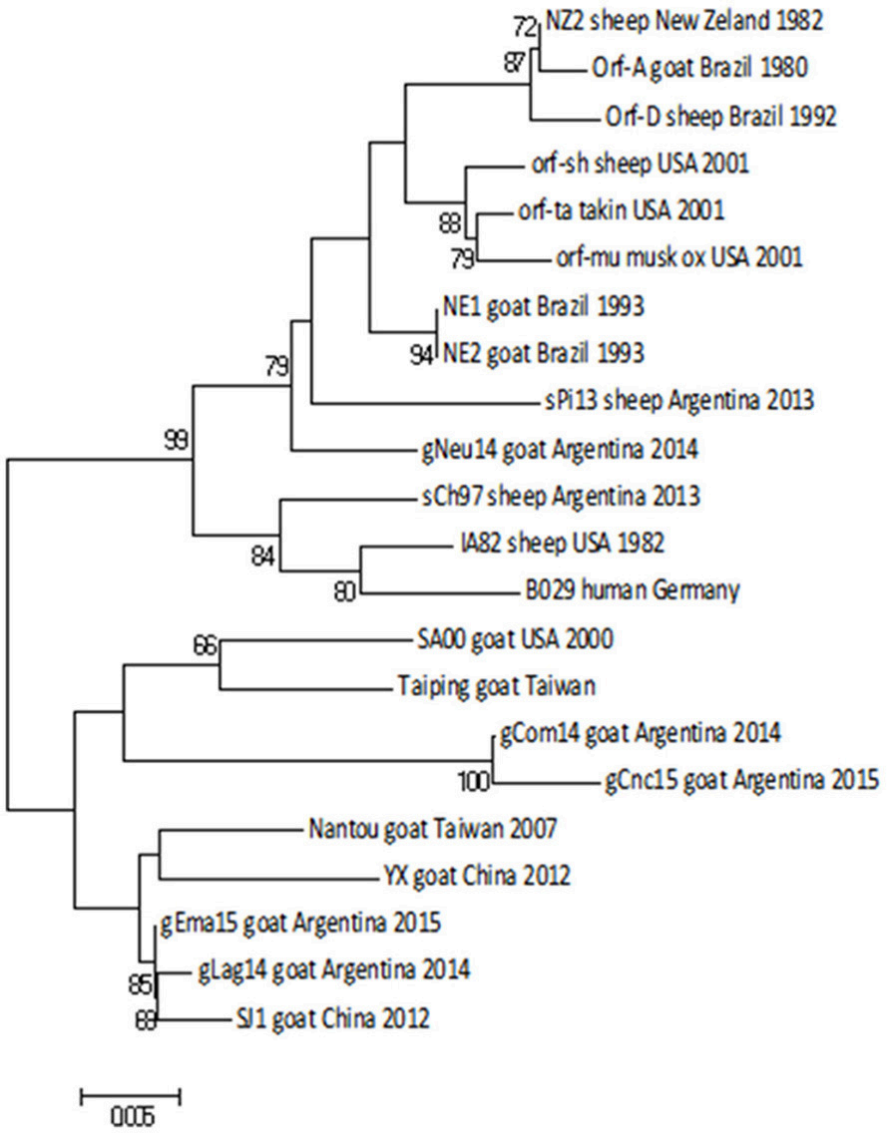
Phylogenetic analysis based on nucleotide sequence of *orf020*. The phylogenetic relationship was constructed by the Neighbor-Joining algorithm using MEGA 6.0 software. Numbers at nodes represent % of one thousand bootstrap replicates. The scale bars are expressed in relative nucleotide sequence difference.

The NJ phylogenetic tree shows that the strains gCom14 and gCnc15 are grouped in the same branch, whereas the two strains from Salta province (gLag14 and gEma15) are together in a different branch of the tree. Finally, the gNeu14 formed a group with Argentinian sheep strains sPi13 and sCh97 with a bootstrap confidence value of 99% (Figure 5).

When we analyzed the *orf127* (vIL10), another virulence gene, the range of nucleotide identity among all Argentinian strains (from 95.2 to 100%) was just slightly higher than the value of variance among all strains known in Gene Bank (94.8–99%).

The gCom14, gCnc15, and gEma15 strains are 100% identical in this gene, whereas gNeu14 presents the largest difference within all Argentinian strains.

**Figure 6:**
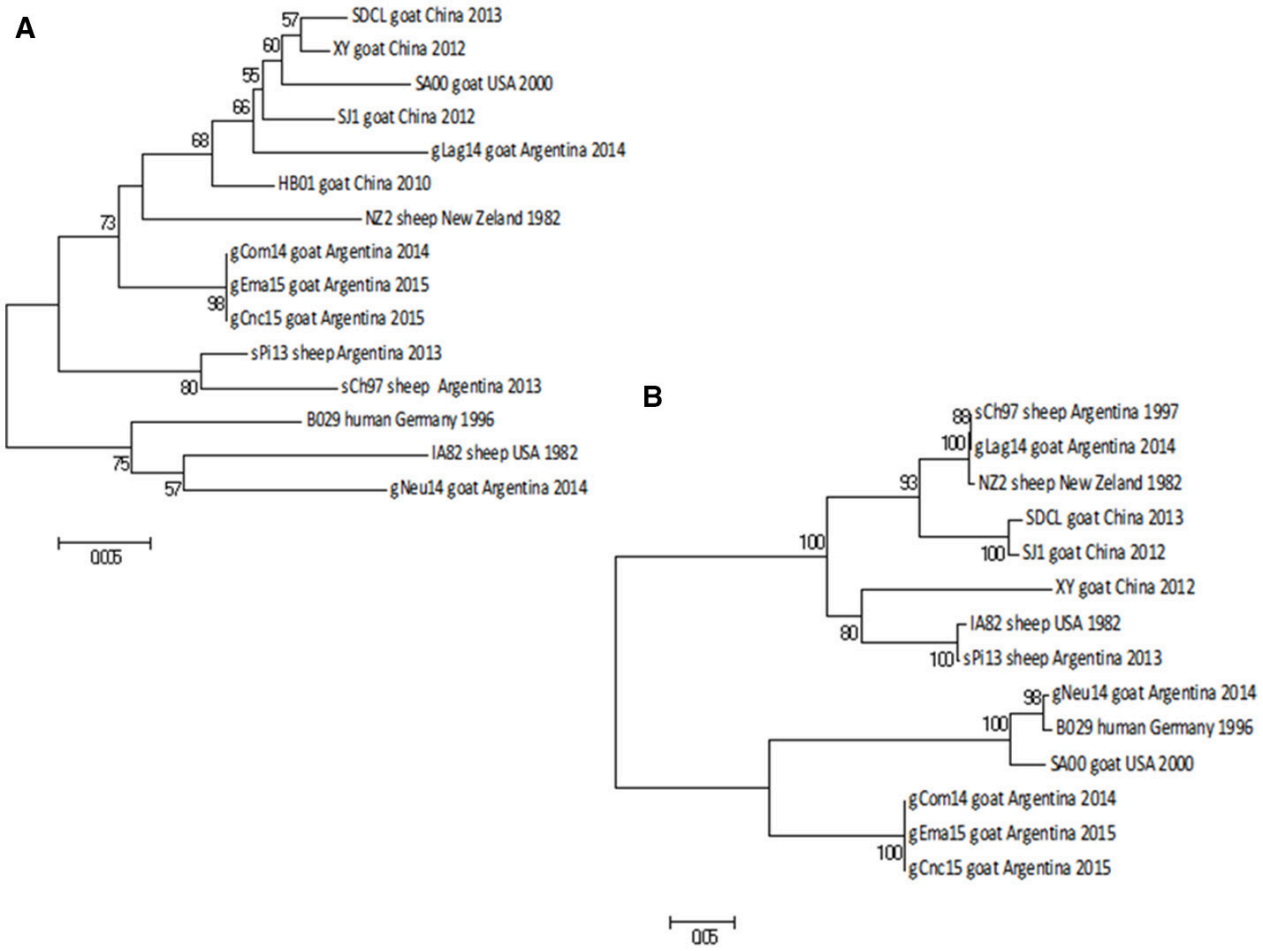
Phylogenetic analysis based on nucleotide sequence of *orf127* and *orf109*. The phylogenetic relationship was constructed by the Neighbor-Joining algorithm using MEGA 6.0 software. Numbers at nodes represent % of one thousand bootstrap replicates. The scale bars are expressed in relative nucleotide sequence difference. **(A)** NJ tree based on *orf127* was obtained with Kimura-2P evolution model. **(B)** NJ based on the sequence of *orf109* was obtained with Tamura-3P evolution model.

As expected, the NJ phylogenetic tree shows that gCom14, gEma15, and gCnc15 are in the same branch (Figure 6A) and formed a big group with other Argentinian strains (gLag14, sPi13, sCh97) with a bootstrap confidence value of 88%.

The *orf109* gene encoding an envelope mature protein (EEV) and is one of the most variable genes within the PPV genus (29, 30). The nt identity among Argentinian strains was from 57.9 to 100%, whereas the identity range among all known strains is from 57 to 99.6%.

As we described with the previous gene, gCom14, gCnc15, and gEma15 are again identical in nucleotide sequence and the strain gNeu14 presents the largest difference within all Argentinian strains.

The NJ phylogenetic tree for *orf109* sequences shows that gCom14, gCnc15, and gEma15 form a homogenous group. The gLag14 strain is closely related to the Argentinian sheep strain sCh97, whereas gNeu14 is related to the B029 and SA00 strains from Germany and USA respectively. All tree branches are highly supported by the bootstrap methodology (Figure 6B).

### Recombination analysis

**Figure 7:**
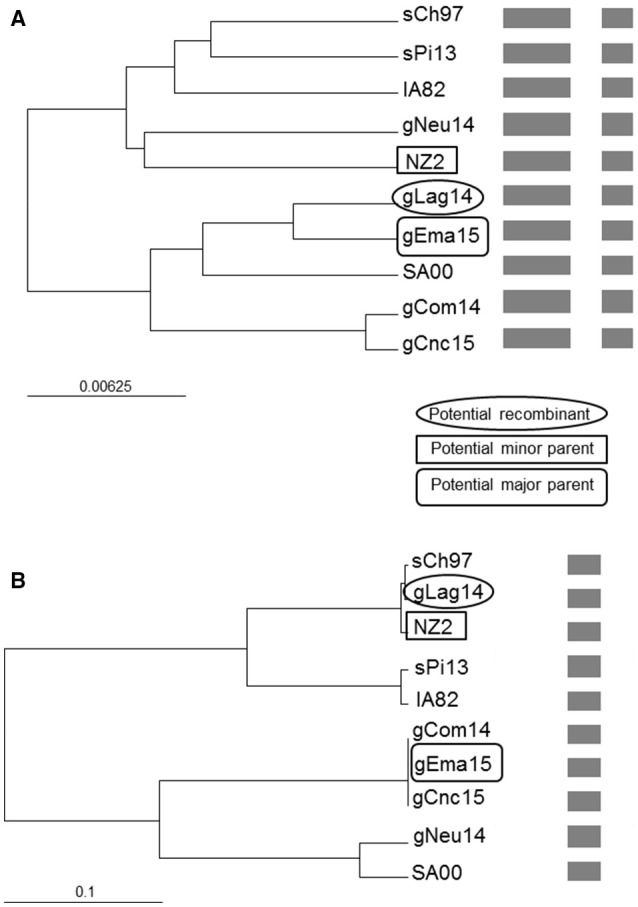
Phylogenetic recombination evidence. UPGMA trees regions derived from **(A)** major parent (1–1,079 and 1,590–2,085) and **(B)** minor parent (1,080–1,589). A schematic representation of the alignment is presented after the names of the isolates. The scale bars are expressed in relative nucleotide sequence difference.

As we found discrepancies among the generated trees from the different genes, we performed a recombination analysis with RDP4. In a preliminary scan, 10 recombination signals were detected in the alignment and, after refining the preliminary hypothesis, two of them showed the strongest support. Due to the limited available sequences, this work goal is not to perform a comprehensive analysis but show the possibility of occurrence. Under this framework, gLag14 was found to be a probable recombinant whose parents were related to gEma15 and NZ2, being *orf109* the recombinant segment (Figure 7 and Figure S1). A second supported recombination event can be found involving gEma15, gConc15, and SA00. In this case, the last two (or the first two) segments are implicated and it is not clear who is the recombinant (Figure S2).

## Discussion

This study presents the first molecular characterization of five CE outbreaks in goats from Argentina.

Although the disease could be readily diagnosed by clinical signs, confirmation was based on electron microscopy and PCR studies. Electron microscopy showed the oval-shaped virion particle, with a helical structure typical of parapoxviruses. Then, we confirmed the clinical diagnosis of CE through the amplification of *orf045* gene encoding the late transcription factor (VLTF-1), which is a highly conserved gene from the Parapoxvirus genome. All samples were positive to this confirmatory ORFV PCR.

To determine if one or several strains caused these outbreaks in herds of goats, we carried out a phylogenetic analysis based on the study of four molecular markers corresponding to four different genes.

The *orf011*gene internal region (nt 388–981), which has been the main target for the phylogenetic analysis for the last years, shows that all Argentinian strains form a heterogeneous group supported by a node with a bootstrap confidence value of 73% (Figure 3). We also observed the formation of two large groups: one of them containing goat strains exclusively, where four of our strains are grouped (gLag14, gCom14, gCnc15, gEma15) and another group consisting mainly of ORFV strains from sheep, where our gNeu14 is located. The same kind of clusters was observed by Schmidt et al. (14), when analyzing seventeen strains of Brazil. Focusing on the amino acid alignment, we could appreciate that ORFV strains from sheep have serine at position 249 (Figure 4), which was described by Kumar et al. (28) as species marker. However, we found in this same group, ORFV strains from goats, takin, and musk-ox that also have S 249. It could be hypothesized that these animals acquired the virus from a sheep. As other researchers observed in previous studies (17, 27, 31), many of the branches of the *orf011* phylogenetic tree have nodes with low or intermediate bootstrap values. This effect could be caused by the high level of identity observed within these samples which result in low phylogenetic signal among ORFV from different countries and continents (14, 26, 32, 33). Due to this low variability and lack of topology resolution, we decided to confirm the phylogenetic relationships using the Parsimony method (data not shown). We obtained no differences in tree topology or branch support and therefore we studied other viral genome regions to deepen the understanding of the phylogenetic relationship among the sequenced viruses.

We analyzed the *orf020* and *orf127* genes, which codify virulence factors VIR and vIL10 respectively, and *orf109* gene because it is one of the most variable genes of the PPV genus according to the comparison of reference genomes (29, 30). The variability is not only given by synonymous and non-synonymous changes of nucleotides but also by differences in the number of codons.

A first remarkable conclusion of this work is that the gCom14 strain that produces the outbreak in Comallo place is different from the strain that caused an outbreak in sheep the previous year (sPi13) in Pilcaniyeu, a place located near Comallo and within the same agroecological area place (see Figure 1). This can be explained because the outbreak in the Comallo place was originated after the introduction of asymptomatic animals coming from another province.

It is important to note that in three out of five cases analyzed in this study, Ecthyma outbreaks began after the introduction of apparently asymptomatic animals. This highlights the importance of knowing the health status of animals traded between farms, in order to avoid the strain dispersion through different territories.

Regarding the gNeu14 isolate, the analysis of the virulence gene *orf020* showed that it is related to sPi13 and near to sCh97 (both Argentinian sheep strains) as described in the analysis of the internal region of *orf011*, and has a bootstrap supported node of 99% (Figure 5). On the other hand, when we analyzed two other genes (*orf127* and *orf109*) (Figures 6A,B) the phylogenetic analysis showed that the gNeu14 strain is phylogenetically distant from other Argentinian strains, despite the remarkable nucleotide difference variation between both genes (3–5 and 25–42%, respectively).

Surprisingly, the gCom14, gCnc15, and gEma15 strains are 100% identical regarding the sequence of the *orf109* gene. This had only been observed when analyzing intra-herd samples (unpublished laboratory data). This study reports for the first time this finding in samples from locations separated by more than 900 km (see Figure 1) and with outbreaks occurring at different months.

The gCom14 and gCnc15 strains are very similar in all four molecular markers analyzed, whereas gEma15 is identical to gCom14 and gCnc15 but only in *orf109* and *orf127* sequences, both genes near to the 5′ end of ORFV genome. When we analyze the markers close to the 3′ end of the genome (internal region of *orf011* and complete *orf020*), the phylogenetic relationships of gEma15 with respect to gCom14 and gCnc15 varies. This could be explained by one or more recombination events on the viral evolution and we found evidence of this phenomenon in our study for some of the samples (gEma15, gConc15, and gLag14) although a more comprehensive analysis must be undertaken to confirm this hypothesis. Besides, other researchers have found possible signs of recombination within the genus Parapoxvirus that could explain divergences or inconsistencies in the phylogeny (34, 35).

The analysis of the four molecular markers used in this study of five different outbreaks, reveals not only the circulation of several strains in Argentina but also the possibility of recombination between them. The fact that the range of nucleotide differences among Argentinian strains, in most of the genes, is comparable with worldwide isolates supports a long-term virus evolution and/or multiple virus entries to the country. However, the number of molecularly characterized strains must be increased to carry out a deep analysis with a greater number of molecular markers or with complete genome sequences to better support these conclusions.

Availability of sequence data from different regions and countries would not only help in updating the knowledge of the scientific community on Contagious Ecthyma disease but also would be highly beneficial in designing suitable polyvalent vaccines with a wider livestock coverage and in formulating appropriate measures for disease control or eradication.

## Author contributions

AP, CAR, and GK contributed conception and design of the study. AP performed diagnostic PCR, electron microscopy and the PCRs for the four molecular markers. CAR, JM, CER, AM, and AC characterized the outbreaks in the field, evaluated the animals, took epidemiological data and took the scab samples. AP and GK performed the phylogenetic analysis. All authors contributed to manuscript revision, read and approved the submitted version.

### Conflict of interest statement

The authors declare that the research was conducted in the absence of any commercial or financial relationships that could be construed as a potential conflict of interest.
