# Supplementary figures and images for: Phylogenetic Analysis of ORF Viruses From Five Contagious Ecthyma Outbreaks in Argentinian Goats
Source: Front Vet Sci. 2018 Jun 19;5:134. doi: 10.3389/fvets.2018.00134 (PMC6018470; doi:10.3389/fvets.2018.00134)

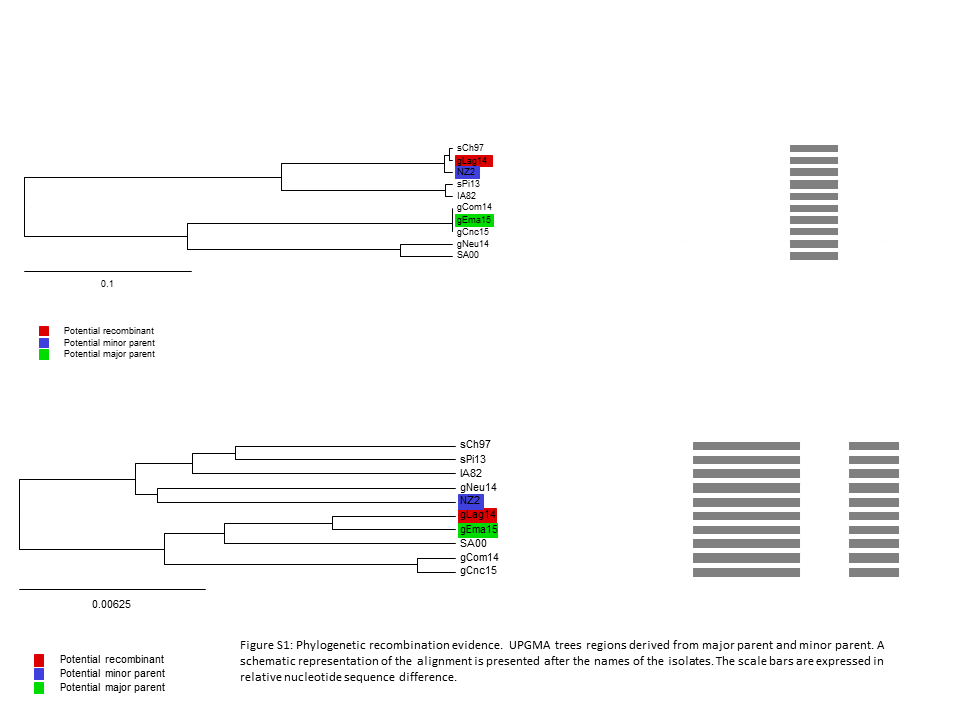

Supplement: Supplementary file 1 [file Image_1.tif]

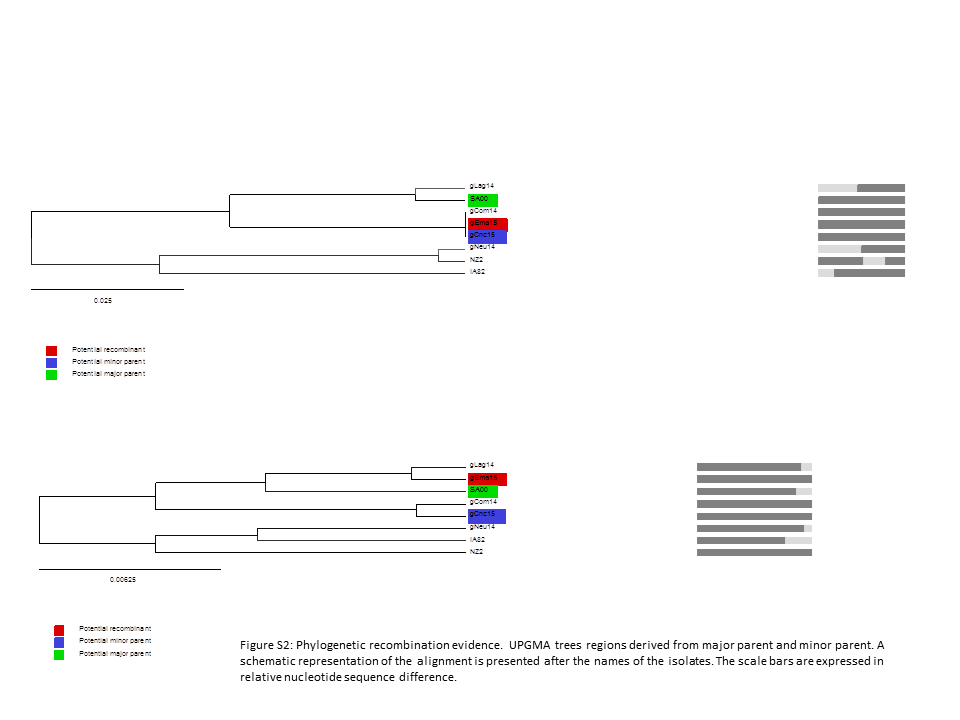

Supplement: Supplementary file 2 [file Image_2.tif]

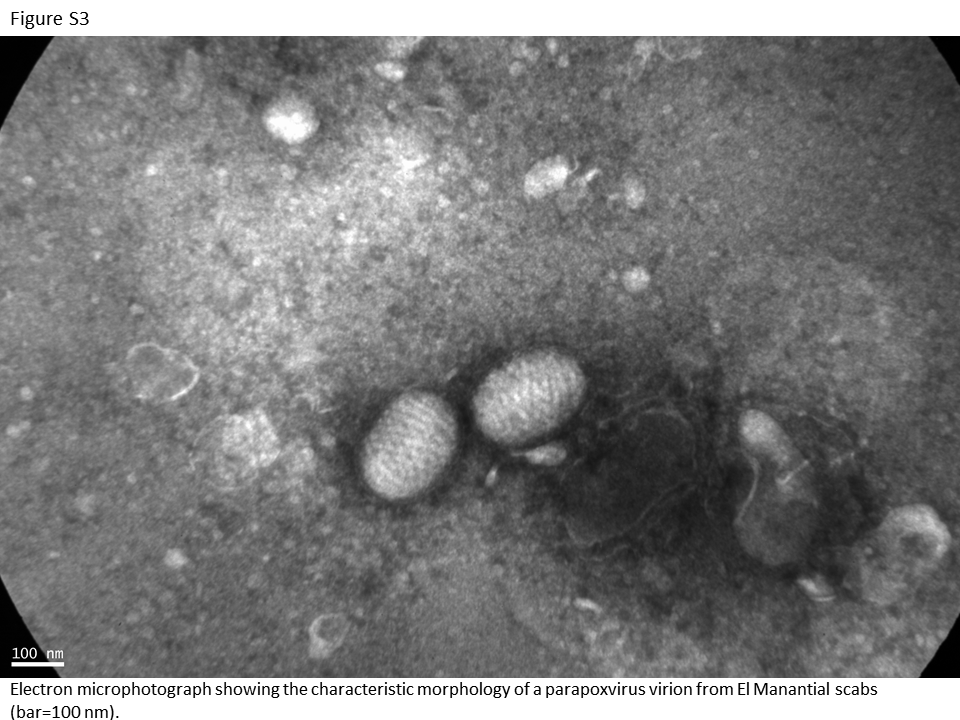

Supplement: Supplementary file 3 [file Image_3.tif]

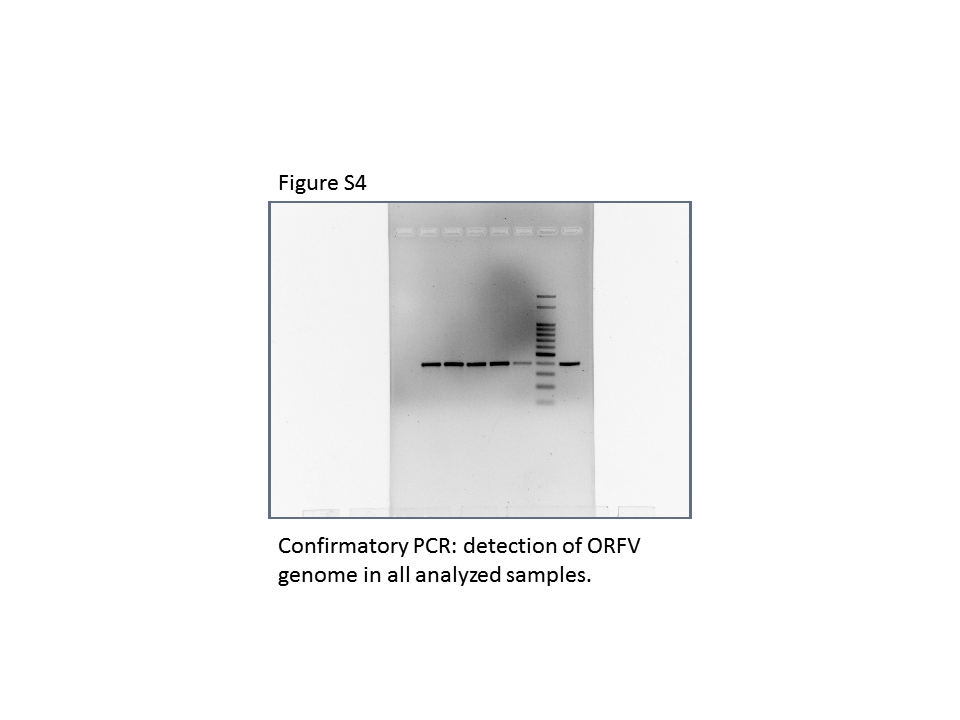

Supplement: Supplementary file 4 [file Image_4.tif]
